# Development and validation of machine learning classifiers for predicting treatment-needed retinopathy of prematurity

**DOI:** 10.1186/s12911-025-03057-w

**Published:** 2025-07-01

**Authors:** Nasser Shoeibi, Majid Abrishami, Seyedeh Maryam Hosseini, Mohammad-Reza Ansari-Astaneh, Razieh Farrahi, Bahareh Gharib, Fatemeh Neghabi, Mojtaba Abrishami, Mehdi Sakhaee, Mehrdad Motamed Shariati

**Affiliations:** 1 Eye Research Center, Mashhad University of Medical Sciences, Mashhad, Iran; 2 Department of Health Information Technology, Ferdows Faculty of Medical Sciences, Birjand, Iran; 3 Eye Research Center, Khatam Al-Anbia Eye Hospital, Gharani Boulevard, Mashhad, Iran

**Keywords:** Artificial intelligence, Machine learning, Prediction models, Retinopathy of prematurity, Treatment

## Abstract

**Background:**

This study aims to design and evaluate various supervised machine-learning models for identifying premature infants who require treatment based on demographic data and clinical findings from screening examinations.

**Methods:**

We conducted a retrospective review of medical records for infants screened for retinopathy of prematurity (ROP) at our clinic over the past decade. We extracted demographic and clinical data, including eleven features: sex, maternal education, paternal education, birth weight, gestational age, ROP stage, zone of retinal involvement, age at examination, weight at examination, and CPR. We developed and assessed several classifiers: logistic regression (LR), decision tree (DT), support vector machine (SVM), naïve Bayes (NB), K-nearest neighbors (KNN), XGBoost, artificial neural networks (ANN), and random forest (RF). The target variable was defined as whether the neonate received any treatment during the follow-up period.

**Results:**

Our analysis included data from 9,692 infants. Among the machine learning models evaluated, the XGBoost and ANN models achieved the highest accuracy at 96%. In terms of sensitivity (recall), the NB model exhibited the lowest false negative rate, indicating the highest sensitivity (0.99). In the context of premature neonates, accurately diagnosing those who require treatment is crucial. Therefore, from a clinical perspective, prioritizing a model with the lowest false negative rate may be more beneficial than selecting one based solely on the highest accuracy.

**Conclusion:**

While AI can enhance decision-making processes by providing real-time risk assessments, these tools must be used to augment—not replace—clinical judgment. Clinicians must remain involved in interpreting model outputs and making final treatment decisions based on a holistic understanding of each patient’s unique circumstances.

**Clinical trial number:**

Not applicable.

## Background

Retinopathy of prematurity (ROP) is a retinal vascular maturation disorder that can lead to irreversible vision loss if left untreated [1]. As the quality of health care in the neonatal intensive care units (NICUs) improves and the survival rate of premature infants increases, the prevalence of ROP is also rising [2]. Approximately 1.2 million preterm newborns in high-income countries exhibit improved survival rates, particularly at lower gestational ages (GA) [3]. ROP is a preventable condition, and timely diagnosis and treatment significantly reduce the risk of visual disabilities in at-risk infants. Each year, premature infants are born in developing and middle-income nations [4]. However, the expansion of ROP screening programs has not kept pace with advancements in newborn care.

Currently, there are locally adapted protocols for screening premature infants at risk of retinopathy of prematurity (ROP) in various regions around the world. In the United States, Retinal screening examinations should be conducted for infants who weigh less than 1500 g at birth or whose gestational age is 30 weeks or less. Additionally, a subset of infants weighing between 1500 and 2000 g at birth or whose gestational age exceeds 30 weeks may also require screening if they exhibit an unstable clinical course, including those who need cardiorespiratory support and who those deemed to be at high risk for ROP by their attending pediatrician or neonatologist [5, 6]. In contrast, the ROP screening protocol in Iran identifies neonates with a gestational age of 34 weeks or less or a birth weight of 2000 g or less as candidates for ROP screening examinations.

A comprehensive retinal examination with dilated pupils should be performed for screening. The timing of follow-up examinations is determined by the severity of the findings observed during the initial assessment. Indirect ophthalmoscopy or RetCam examination is utilized to evaluate the retina [7, 8].

Most artificial intelligence studies in Retinopathy of Prematurity (ROP) have focused on diagnosis, prediction, and prognosis based on retinal imaging findings in neonates [9]. Research utilizing non-imaging data and demographic information for diagnosis and prognosis in ROP is notably limited. Today, the application of machine learning methods as clinical decision-support systems in ophthalmology is expanding significantly. The increasing availability of complex clinical datasets has led to a steady rise in the use of machine learning (ML) in clinical research [10]. Machine learning offers substantial advantages in prognostic performance and identifying previously unrecognized patient subpopulations with specific physiologies and prognoses. Additionally, the growing use of telemedicine in ophthalmology has prompted new studies focused on utilizing artificial intelligence (AI) to screen infants at risk of ROP [11, 12].

This study aimed to design and compare various supervised machine-learning models to detect premature infants requiring treatment based on demographic data and clinical findings from the screening examination.

### Novelty, aim, and objectives

- **Novelty**: This study provides a comparative analysis of multiple machine learning classifiers for predicting treatment-needed retinopathy of prematurity (ROP) using a clinically relevant dataset. It is one of the few studies to focus on the internal validation of models and emphasize sensitivity as a crucial metric in a high-stakes medical setting.
- **Aim**: The primary aim of this study is to develop, validate, and compare machine learning models to improve the prediction of treatment-needed ROP in neonates, with an emphasis on minimizing false negatives (missed diagnoses).

### Objectives

- Conduct exploratory data analysis to understand the dataset and determine key correlations.
- Evaluate the performance of machine learning classifiers, including logistic regression (LR), decision tree (DT), support vector machine (SVM), naïve Bayes (NB), K-nearest neighbors (KNN), XGBoost, artificial neural networks (ANN), and random forest (RF), with a focus on both accuracy and sensitivity.
- Identify a model that balances clinical relevance, ensuring sensitivity for treatment-needed cases while maintaining acceptable specificity.
- Discuss the implications of the model’s performance in the context of real-world clinical decision-making and future improvements.

The remainder of this paper is structured as follows: In the “Methodology” section, we describe the dataset used, the preprocessing steps, and the machine learning algorithms applied. The “Results” section is divided into two parts: First, it presents the findings from exploratory data analysis and correlation analysis to give a comprehensive overview of the relationships between key variables, followed by a detailed evaluation of the performance of the machine learning models. In the “Discussion”, we analyze the clinical implications of the model results and offer insights into potential future directions.

## Methods

**Fig. 1:**
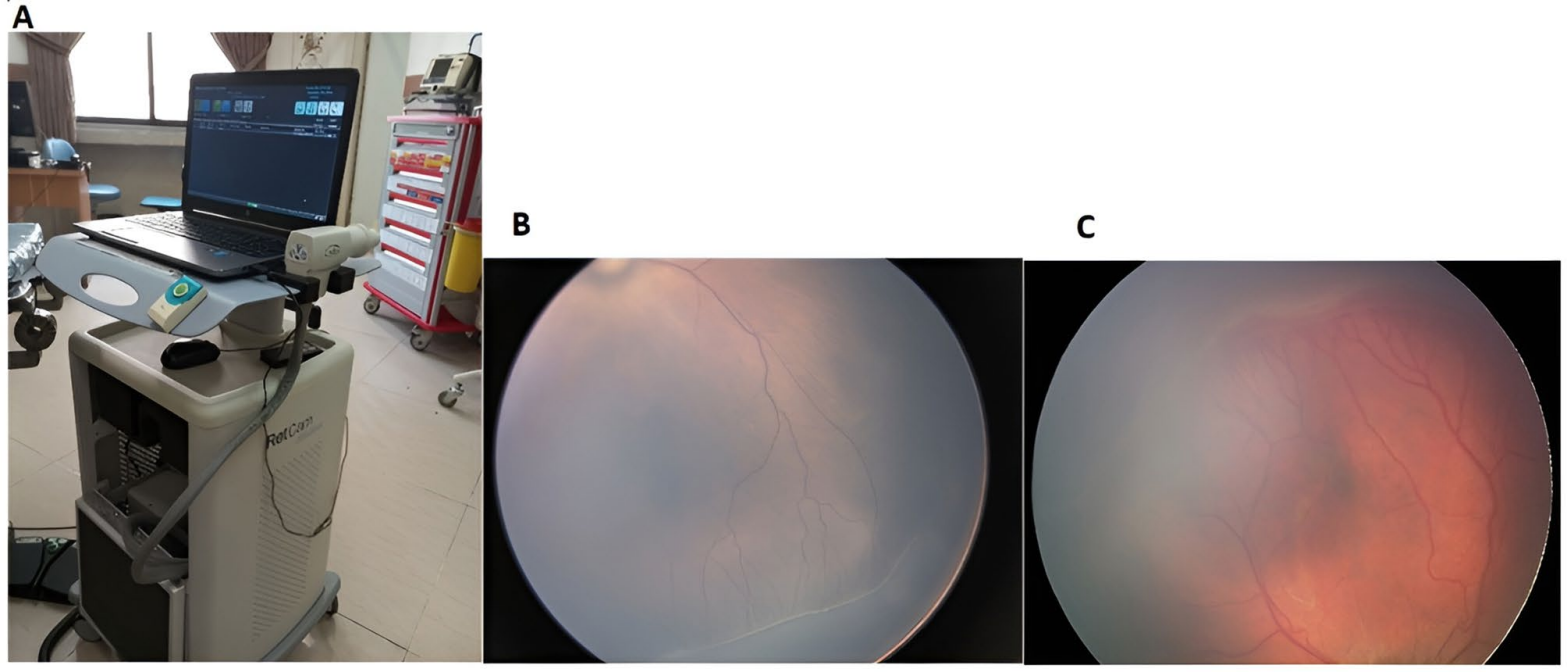
**A**: The RetCam fundus imaging device utilized in the ROP clinic at Khatam Al-Anbia Eye Hospital. **B**: A representative image of a neonate with retinopathy of prematurity (ROP) exhibiting stage 2, zone 2 involvement. **C**: A representative image of a neonate with ROP demonstrating stage 3 involvement in zone 2

The data utilized in this study were extracted from the electronic medical records of premature infants examined in the ROP clinic at Khatam Al-Anbia Eye Hospital, Mashhad, Iran. The sample size was determined based on the availability of all eligible newborns screened for ROP at our university hospital between 2020 and 2024. Given the study’s machine learning approach, a large dataset was beneficial for model training and validation. A non-probability consecutive sampling method was used, including all preterm neonates who met the inclusion criteria during the study period. This ensured comprehensive data collection without selection bias. Premature infants requiring screening examinations for ROP following the national protocol are referred to this center. The ROP center at Khatam Al-Anbia Hospital is the only facility dedicated to ROP treatment in eastern Iran. Standard image acquisition is performed by a trained nurse using the RetCam device (Fig. 1). A pediatric retina specialist reviews all images. If further evaluations are deemed necessary, an indirect ophthalmoscopy is performed. Demographic data and clinical findings are meticulously recorded in the patient’s electronic medical record (EMR). The primary outcome of this study was the presence or absence of treatment-needed retinopathy of prematurity (TN-ROP), defined based on international classification criteria. TN-ROP was determined by experienced ophthalmologists according to established screening guidelines.

### Study features (Domain experts’ Recommendations)

**Fig. 2:**
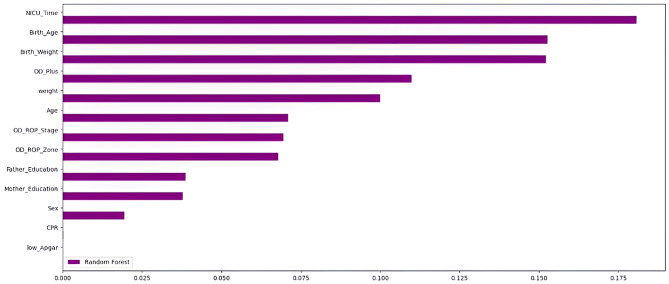
Feature importance analysis with the Random-Forrest method

In this study, we reviewed the medical records of infants screened for retinopathy of prematurity (ROP) at the clinic over the past ten years. Demographic data and clinical findings from the screening examination were extracted. Predictive features (the feature vector) were selected based on the recommendations of domain experts. Additionally, we employed the Random Forest model as our method for feature importance analysis (Fig. 2). Chi-square tests and t-tests were employed to compare categorical and continuous variables, respectively. Normality was assessed using the Shapiro-Wilk test, and Levene’s test was used for homogeneity of variance. Non-parametric alternatives were used when assumptions were violated. Statistical analyses and machine learning modeling were conducted using Python 3.8 (open-source), Scikit-learn, Shapley additive explanations (SHAP), and SPSS (version 11.5).

All features were deemed for inclusion in model training (Table 1), except the low-Apgar. The predicted variable (target label) was defined as whether the neonate received any treatment during the follow-up period (Table 2). The dataset used in this study was compiled from records of 9,692 infants, each of whom underwent screening for retinopathy of prematurity (ROP). The data were organized into a structured spreadsheet format (Excel) to facilitate analysis and included 12 key features relevant to the clinical prediction of treatment-needed ROP. These features were: Sex, Mother’s education, Father’s education, History of CPR, Birth age, Birth weight, NICU time of hospitalization, Age, weight, ROP stage, ROP zone, and presence of plus disease. The dataset provides a comprehensive overview of the infants’ clinical and demographic information, which was crucial for building and validating the machine learning models used in this study. Qualitative data (e.g., presence of TN-ROP, gender, mode of delivery) were summarized using frequencies and percentages (%). Quantitative data (e.g., birth age, birth weight, NICU duration) were reported as mean ± standard deviation (SD) for normally distributed variables and median (interquartile range) for non-normally distributed variables.

**Fig. 3:**
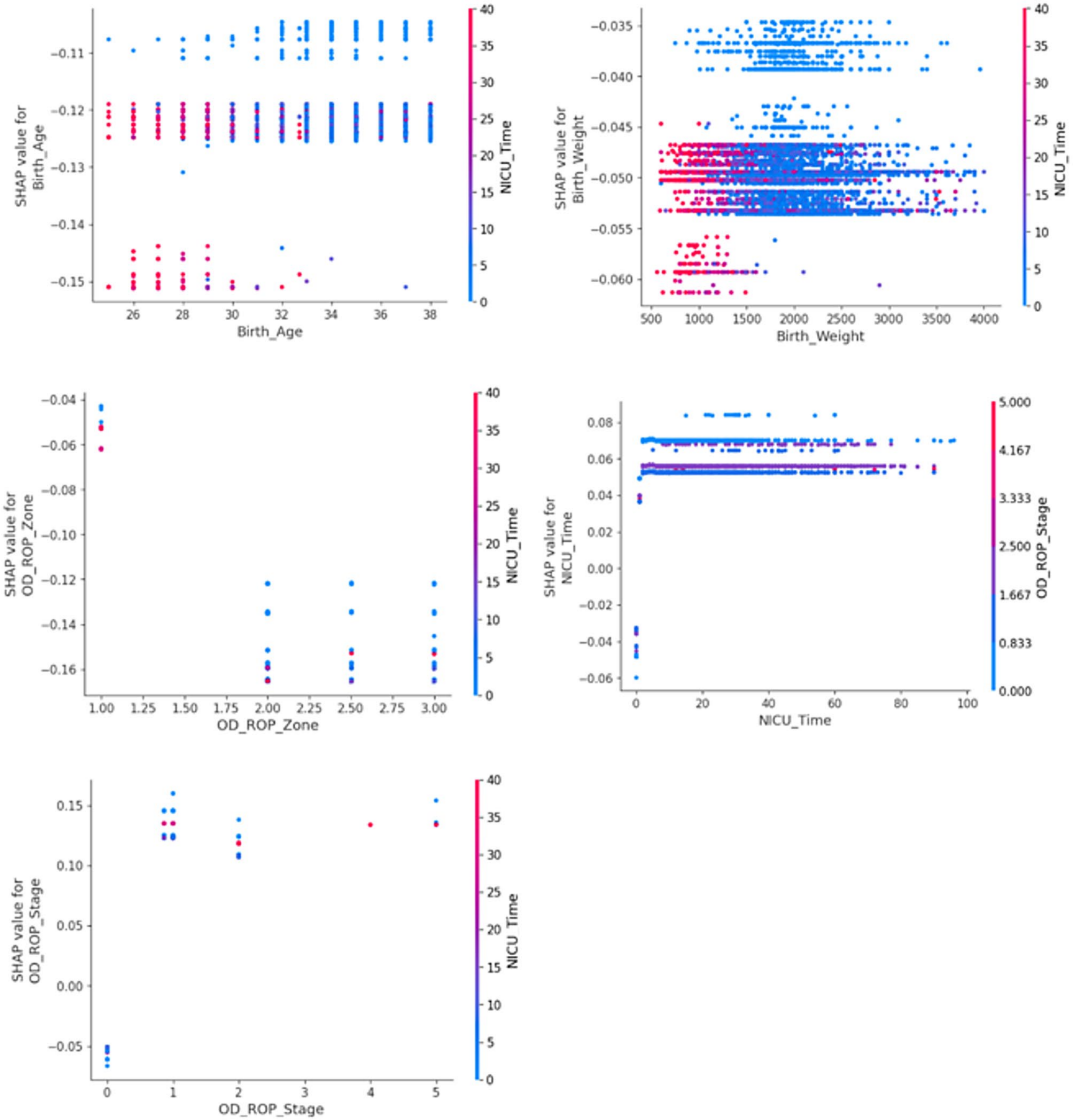
The SHAP dependence plots illustrate the relationship between key predictors and treatment-needed risk. Prematurity (lower birth age), low birth weight, and more extended NICU stays are strongly linked to higher model predictions. More severe ROP stages and more central zone involvement (Zone 1) increase risk predictions. The color mapping confirms that these risk factors interact, with NICU time being a critical variable linking birth weight and ROP severity

To enhance the explainability of our random forest model, we applied Shapley Additive Explanations (SHAP) to quantify the contribution of individual features to the prediction of treatment-needed patients (Fig. 3).

**Table 1:** Predictive features of the study

Feature name	Type	Definition and Labeling
Sex	Binary	1: Male, 2: Female
Mother_Education	Ordinary	1: primary school, 2: under diploma, 3: diploma, 4: BSc, 5: MSc, 6: doctorate or more
Father_Education	Ordinary	1: primary school, 2: under diploma, 3: diploma, 4: BSc, 5: MSc, 6: doctorate or more
CPR	Binary	0: No history of CPR, 1: History of CPR
Birth_Age	Continuous	Gestational age at birth
Birth_Weight	Continuous	Body weight at birth
NICU_Time	Continuous	The number of hospitalization days in the NICU
Age	Continuous	The age of the neonate at the time of fundus examination
Weight	Continuous	The weight of the neonate at the time of fundus examination
ROP_Stage	Ordinary	The ROP stage at the time of the first examination
ROP_Zone	Ordinary	The ROP zone of vascularization at the first examination
Plus	Binary	0: No plus disease, 1: Plus disease exists

**Table 2:** Target label

Target (event1)	Definition of labels
0	retinal vascularization completed without any treatment
1	a treatment, either intravitreal bevacizumab or laser photocoagulation, has been utilized for the neonate

### Main phases of the study

**Fig. 4:**
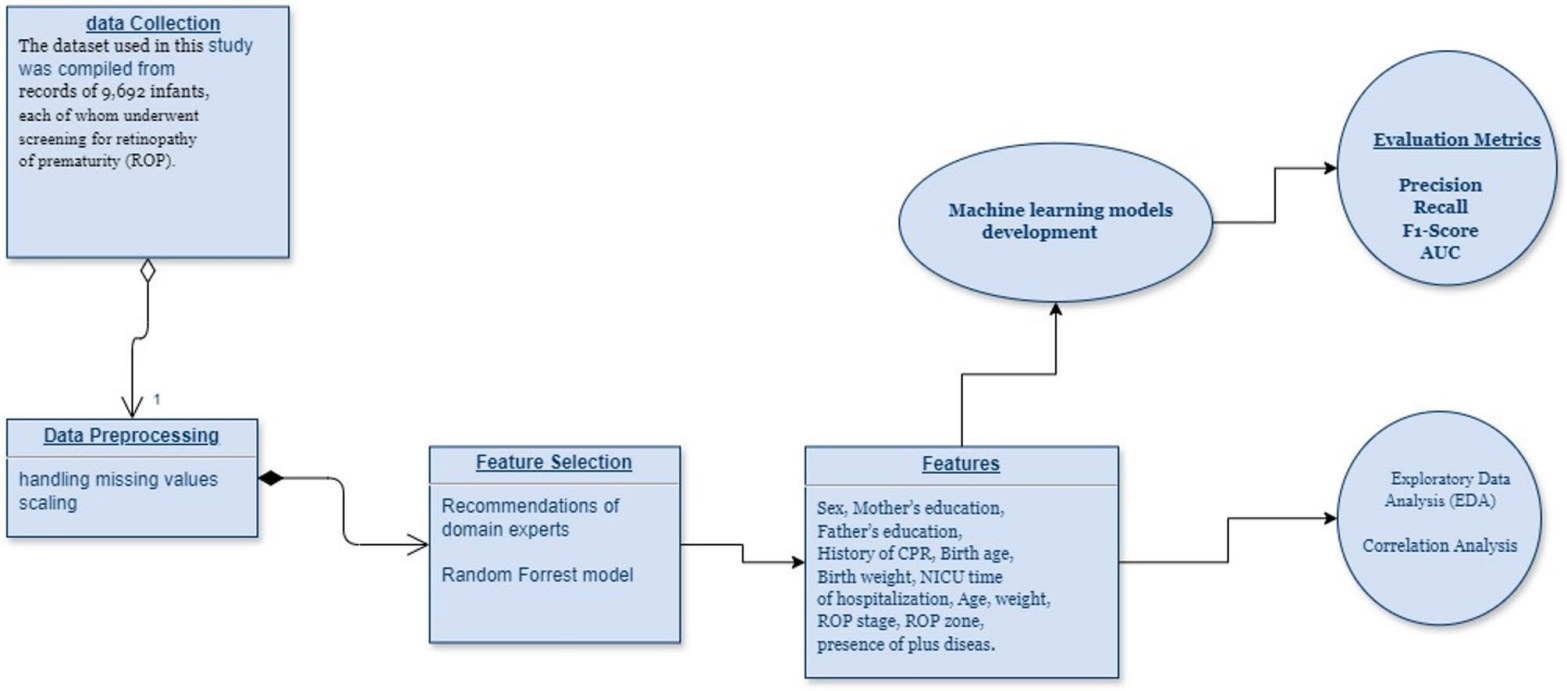
The main phases for developing and evaluating different ML models to predict treatment-needed ROP neonates

We employed descriptive statistics and correlation analysis to explore the data. Data preprocessing included handling missing values via mean/median imputation and standardizing continuous variables. Outliers were detected using Z-scores (> 3 SD from the mean) and the inter-quartile range (IQR) method (values beyond 1.5*IQR). Selected features were utilized to train machine-learning models. Given the target variable is binary, we implemented classification methods. The classifiers designed and evaluated in this study included logistic regression (LR), decision tree (DT), support vector machine (SVM), naïve Bayes (NB), K-nearest neighbors (KNN), XGBoost, artificial neural networks (ANN), and random forest (RF) (Fig. 4). We used 5-fold cross-validation to ensure generalizability and internal validation to evaluate model robustness. We optimized hyperparameters using Random Search with Cross-Validation for simpler models and Bayesian Optimization for complex models like XGBoost and neural networks. The best hyperparameters were selected based on the highest AUC-ROC on the validation set.

A diverse range of machine learning (ML) classifiers were evaluated in this study to explore the balance between model complexity, predictive performance, and interpretability in predicting treatment-needed retinopathy of prematurity (ROP). The selection of models was guided by the need to benchmark both traditional and advanced algorithms across the characteristics of our dataset, which included a mix of structured clinical variables, potential non-linear relationships, and class imbalance. We included both interpretable models suitable for clinical translation and more sophisticated models capable of capturing complex data patterns. Including simpler models like LR and NB offers transparency and interpretability, which are desirable in clinical decision-making. Tree-based models (DT, XGBoost) were chosen for their capacity to handle non-linear relationships and interactions among clinical variables. XGBoost, though computationally intensive, is robust and effective in overfitting imbalanced datasets — a key feature of our clinical data where treatment-needed cases are a minority. The ANN was included to assess whether capturing complex, non-linear feature interactions could yield better performance, recognizing its potential trade-off in interpretability and computational cost. This diverse model selection allowed for a comprehensive evaluation of the trade-offs between model accuracy, complexity, and clinical applicability.

### Evaluation metrics

To evaluate the model performance, we used the following metrics:

- **Accuracy**: The proportion of correct predictions made by the model out of all predictions.
- **Sensitivity (Recall)**: The ability of the model to correctly identify true positive cases (infants requiring treatment for ROP).
- **Precision**: The proportion of true positive cases among all cases predicted as positive by the model.
- **F1-Score**: A balanced measure that combines precision and recall.
- **AUC-ROC**: A measure of the model’s ability to distinguish between positive and negative cases, providing insight into overall classification performance.

## Results

### Exploratory data analysis (EDA)

In this study, data from 9,692 infants were included. The mean gestational birth age and weight of neonates were 32.70 ± 2.52 weeks and 1812.11 ± 501.40 g, respectively. A summary of the descriptive statistics for the variables is presented in Table 3.

**Table 3:** Descriptive statistics of the study variables (feature variables and the target)

Variables	Descriptive statistics
Sex (label: Number)	1: 4553, 2: 5139
Mother_Education (label: Number)	1: 3384, 2: 2280, 3: 319, 4: 1200, 5: 236, 6: 49
Father_Education (label: Number)	1: 3446, 2: 2278, 3:380, 4: 996, 5: 298, 6: 69
CPR (label: Number)	1: 12, 2: 9680
Birth_Age (mean ± sd) (weeks)	32.70 ± 2.52
Birth_Weight (mean ± sd) (grams)	1812.11 ± 501.40
NICU_Time	12.79 ± 12.90
Age (mean ± sd) (weeks)	37.40 ± 2.63
Weight (mean ± sd) (grams)	2434.64 ± 820.00
ROP_Stage (label: Number)	0: 3232, 1: 2496, 2: 2130, 3: 0, 4: 2, 5: 6
ROP_Zone (label: Number)	1: 136, 2: 3647, 3: 4111
Plus (label: Number)	1: 124, 2: 4940
Target variable (event1)	0: 9264, 1: 428

**Fig. 5:**
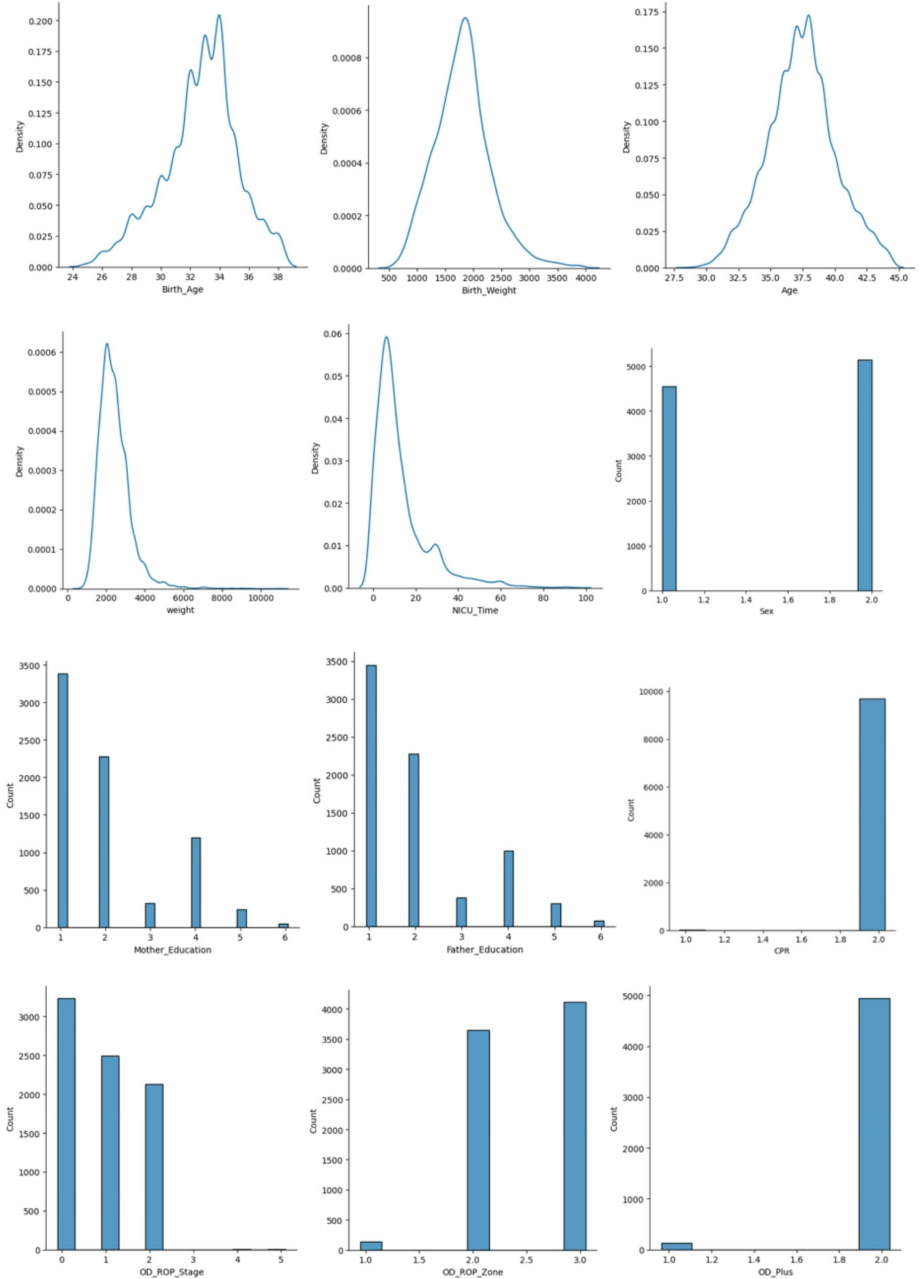
The distribution of features

The distribution of features is summarized in Fig. 5.

**Fig. 6:**
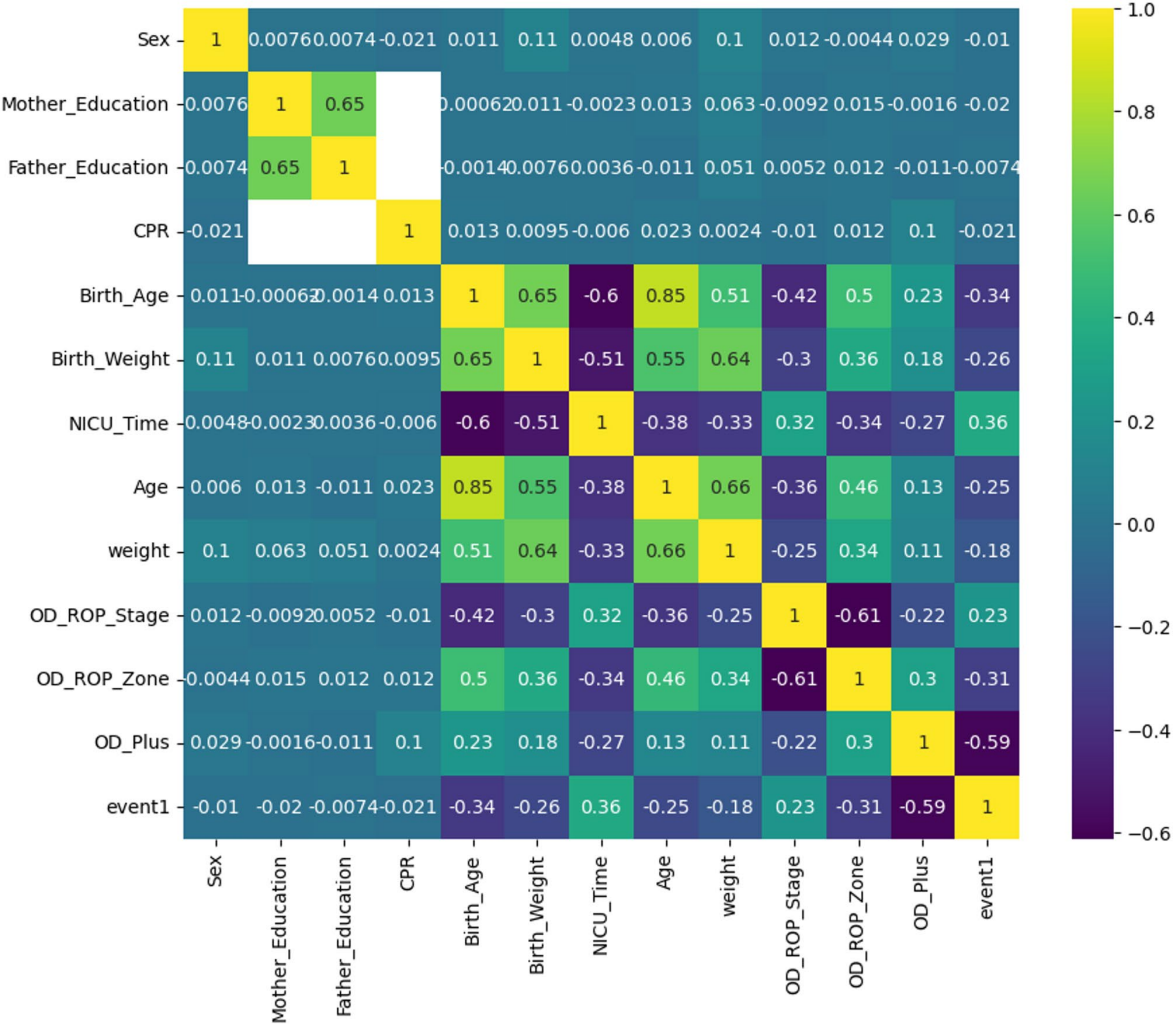
Correlation analysis. The heat map shows the correlation coefficients between variables

Given that the missing data followed a completely random pattern (missing completely at random, MCAR), we employed mean imputation to address the missing values. A Pearson correlation analysis was conducted to explore the relationship between the features and the target variable with the results summarized in Fig. 6.

### Development of machine learning models

The sample was divided into a training set (80%) and a test set (20%) using the train_test_split module. The feature variables were scaled using the StandardScaler preprocessing algorithm. Logistic regression (LR), decision tree (DT), support vector machine (SVM), naïve Bayes (NB), K-nearest neighbors (KNN), XGBoost, artificial neural networks (ANN), and random forest (RF) were developed using the training dataset. Regarding the imbalanced dataset, we implemented the oversampling methods, including the synthetic minority oversampling technique (SMOTE), and adaptive synthetic sampling (ADASYN), to improve classification metrics.

### Evaluation of model performance

Neonates in need of treatment that were correctly predicted were defined as true positive (TP), while those in need of treatment that were incorrectly predicted were classified as false negative (FN). Neonates who did not need treatment and were correctly predicted were classified as true negative (TN), and infants who did not require treatment but were incorrectly predicted as needing it were defined as false positive (FP).

We employed various evaluation metrics, including precision, recall, F1-score, and accuracy to assess the performance of different classification models (Table 4). A classifier’s recall (TP / (TP + FN)) quantifies its ability to find all relevant instances for each class. Precision, defined as TP / (TP + FP), measures the classifier’s ability to correctly identify instances of each class. The F1 score, which is the weighted harmonic mean of recall and precision, is normalized between 0 and 1 (F1 = 2*(Precision*Recall)/ (Precision + Recall)), with a score of 1 indicating a perfect balance between recall and precision.

**Table 4:** Summary of the classification reports metrics

Model		Precision	Recall	F1-score	AUC	Accuracy
KNN					0.77	0.89
	0	0.98	0.91	0.94		
	1	0.27	**0.64**	0.437		
LR					**0.86**	0.84
	0	0.99	0.84	0.91		
	1	0.22	**0.89**	0.36		
NB					0.74	0.61
	0	1	0.49	0.66		
	1	0.09	**0.99**	0.17		
SVM					**0.87**	0.82
	0	0.99	0.82	0.90		
	1	0.21	**0.92**	0.34		
DT					0.70	0.94
	0	0.97	0.97	0.97		
	1	0.37	**0.44**	0.40		
XGBoost					0.73	0.96
	0	0.97	0.99	0.98		
	1	0.63	**0.43**	0.51		
ANN					0.69	0.96
	0	0.97	0.99	0.98		
	1	0.63	**0.43**	0.51		
RF						
	0	0.99	0.85	0.92	**0.87**	0.85
	1	0.24	**0.89**	0.37		

KNN: K-nearest neighbors, LR: logistic regression, NB: naïve Bayes, SVM: support vector machine, DT: decision tree, ANN: artificial neural networks, RF: random forest

**Fig. 7:**
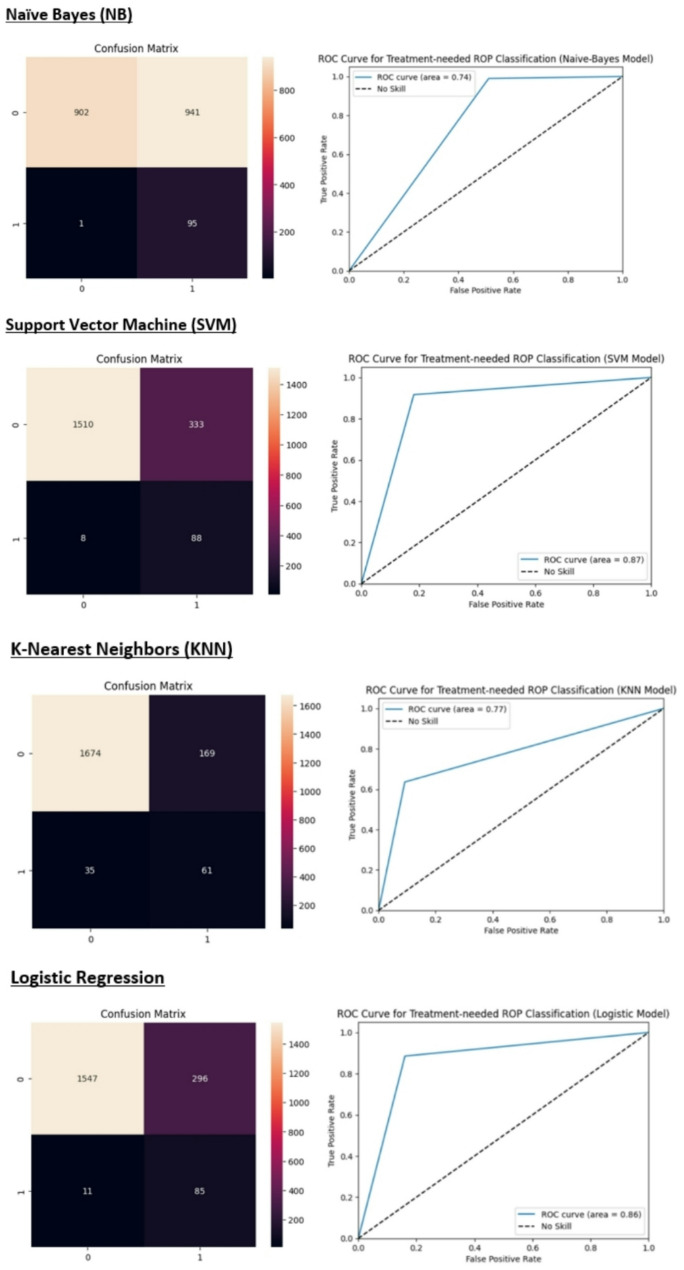

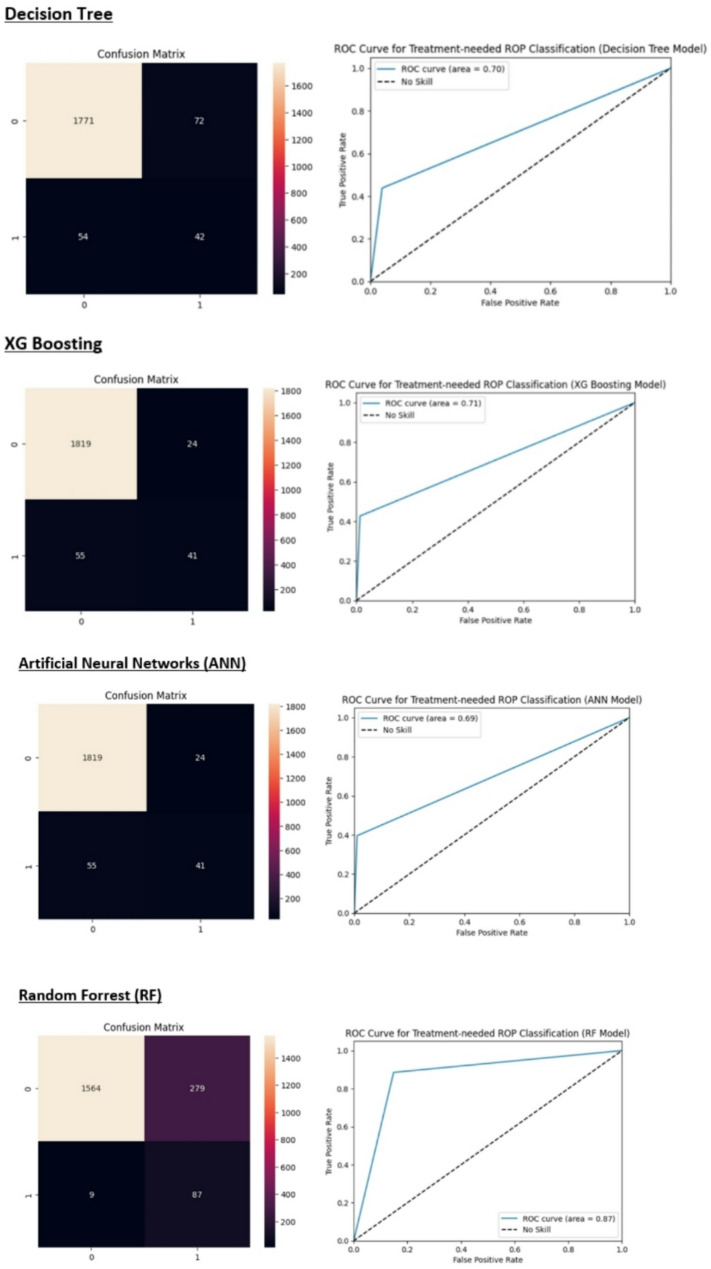
The heatmap of the confusion matrix and the ROC curve for each classifier

Figure 7 summarizes the confusion matrix and the receiver operating characteristic (ROC) curve for each model.

## Discussion

The application of artificial intelligence (AI) and machine learning in healthcare has gained momentum due to its potential to improve diagnostic accuracy and efficiency. In our study, we developed and evaluated several supervised ML classifiers to predict treatment-needed ROP based on demographic and clinical data. The results demonstrated that while multiple models achieved high accuracy rates, sensitivity emerged as a critical factor in their practical application. Although the predictive performance of our model was notably strong in identifying neonates who achieved spontaneous retinal vascularization without requiring treatment, its precision in detecting treatment-needed cases was relatively lower. This trade-off partly reflects the class imbalance in the dataset, with treatment-needed cases being far less frequent. Importantly, the clinical motivation of this model is to reduce unnecessary screening and follow-up visits in low-risk neonates. This strategy aligns with optimizing healthcare resources and minimizing stress for infants and caregivers. From a clinical perspective, a model with high negative predictive value (NPV) and specificity can significantly contribute to safe and efficient triaging in ROP screening programs.

We used eleven features, including sex, mother education, father education, birth weight, birth age, ROP stage, zone of retinal involvement, presence of plus disease, age at the time of examination, weight at the time of examination, and CPR, for prediction. The XGBoost and ANN models achieved the highest accuracy at 96% and the naïve Bayes (NB) model exhibited the lowest false negative rate, indicating the highest sensitivity. While our XGBoost and ANN models achieved a high overall accuracy of 96%, it is essential to interpret this metric with caution in the context of an imbalanced dataset where most neonates do not require treatment for retinopathy of prematurity (ROP). High accuracy in this scenario may obscure poor performance in identifying the minority class, which in this case represents neonates who do require treatment (class 1). Specifically, the model’s sensitivity (recall) for treatment-needed cases was only 43%, indicating that a substantial proportion of true positive cases could be missed. This represents a significant limitation, as false negatives in medical settings can have severe consequences, potentially leading to delays in necessary treatment. However, the NB and SVM models with a recall equal to 99% and 92% for the minority class, showed satisfactory results. Besides, the SVM and RF models showed the highest AUC value among our models (0.87).

Following healthcare improvement in the neonatal intensive care units (NICU) in developing countries, the survival rate of premature neonates has increased. This has led to an increase in the prevalence of ROP in these communities [13, 14]. Given the high stakes involved in ROP diagnosis, there is a pressing need for supplementary tools that enhance the sensitivity of detection methods. Machine learning (ML) models can play a significant role in this regard. By analyzing large datasets and identifying complex patterns that may not be evident to human observers, ML algorithms can assist clinicians in making more informed decisions regarding treatment needs. Considering the models sensitivity, the NB and SVM models in this study showed satisfactory results.

Abnormal neovascularization, which usually takes place in two postnatal phases, characterizes the development and progression of ROP. Neovascular complications lead to blindness in these patients. The occurrence of blindness in a baby brings a lot of psychological and financial costs to the patient’s family and the health system [15–17]. Timely diagnosis of ROP and appropriate treatment including intravitreal injection of anti-VEGF or retinal laser photocoagulation can prevent blindness, which shows the importance of ROP screening in premature neonates [18].

Numerous epidemiological studies have investigated the risk factors involved in associated with the onset and progression of ROP. While the majority of neonates with ROP recover without intervention, those with lower birth weight, lower birth age, extended stays in the neonatal intensive care unit (NICU), more oxygen therapy, more severe ROP at the time of screening examination, and the presence of systemic illness are at a higher risk of requiring treatment [19–21].

We calculated the correlation between the factors which is presented in Fig. 5. The correlation matrix indicates that the predictive features are not highly correlated. In our study, feature importance analysis using the Random-Forrest model revealed that the NICU hospitalization duration, birth age, birth weight, and the presence of plus disease are the most significant predictors for identifying neonates in need of treatment. Anticipating the likelihood of requiring treatment in the future can facilitate the identification of high-risk patients. Consequently, it is advisable to conduct follow-up examinations of these patients at shorter intervals and to provide more comprehensive care regarding weight gain, feeding, and systemic care [22, 23]. When it comes to improving overall performance in machine learning (ML) models, feature selection (FS) is an important method for optimizing data preparation. The use of metaheuristic FS algorithms has significantly increased in recent years. This is explained by their ability to precisely recognize and choose the most relevant features for machine learning tasks. To effectively identify significant features while simultaneously reducing the occurrence of irrelevant ones, simplifying overall complexity, and improving accuracy, Singh et al. present an innovative methodology that makes use of three soft-computing algorithms: Emperor Penguin Optimization (EPO), Gravitational Search Optimization Algorithm (GSA), and their proposed hybrid hGSAEPO. They demonstrated how the categorization of Wisconsin Diagnostic Breast Cancer (WDBC) data sets using these soft computing techniques and six ML classifiers provides a viable framework for prognostic research [24].

We implemented diverse machine learning methods to evaluate and compare their accuracy and sensitivity in predicting treatment-needed retinopathy of prematurity neonates. Regarding the imbalanced dataset, we used the oversampling methods to improve our model performance. The description, strengths, and weaknesses of these methods are presented in Table 5. The performance analysis results of different ML models in this study showed that the XGBoost and ANN models have the highest accuracy (96%). However, the accuracy of KNN, LR, SVM, RF, and DT models is also high (more than 80%). As we showed in Fig. 5, the SVM, RF, and LR models had the highest AUC-ROC (area = 0.87, 0.87. 0.86, respectively) among the models. However, considering the high FP rate, the NB model showed the lowest accuracy (61%). As the results showed, the NB and SVM models exhibited the highest sensitivity, making them potentially more suitable for detecting treatment-needed ROP cases (class 1), despite their lower overall accuracy. In high-stakes medical environments, including neonatal care, where the cost of false negatives is significant, models with higher sensitivity should indeed be prioritized. Ray et al. assessed predicting the incidence of ROP on premature babies on a variety of efficient classifiers including Naive Bayes, Random Forest, and Support Vector Machines (SVMs), and concluded that the highest accuracy (84.36%) achieved, is by 3 layered Hierarchical Committee of Trees (LCT) model, which also outperforms other classifiers in terms of accuracy [25]. The use of machine learning methods and especially deep learning in the diagnosis of diseases in medicine has become popular in recent years. Singh et al. in a study in 2024, compared eight different ImageNet models from Optical Coherence Tomography of Glaucoma to evaluate these models’ performances on various efficiency metrics in glaucoma diagnosis. According to experimental results, VGG16 with the Root Mean Squared Propagation Optimizer achieves 95.68% accuracy and good performance [26]. Recently, the use of medical images such as fundus photographs for the training and evaluation of deep learning algorithms has greatly contributed to the design of clinical decision support systems (CDSS) [27]. A computer-assisted diagnosis (CAD) system based on artificial intelligence was presented by Singh et al. It is designed to offer the best characteristics for machine learning approaches for classifying subject retinal photographs as “healthy” or “sick” in glaucoma patients. The best specificity of 0.9940, sensitivity of 0.9347, and maximum accuracy of 96.55% are demonstrated by the suggested approach [28].

**Table 5:** Classifiers, their strengths, and weaknesses

Classifier	Description	Strength	Weakness
Logistic Regression (LR)	statistical method for predicting binary classes. It uses a logistic function to model the probability of a certain class or event.	Simple and interpretable. Works well with linearly separable data. Less prone to overfitting in low-dimensional spaces.	Assumes linearity between the independent variables and the log odds of the outcome. Not suitable for complex relationships or interactions without transformations.
Decision Tree (DT)	flowchart-like structure where each internal node represents a feature, each branch represents a decision rule, and each leaf node represents an outcome.	Easy to interpret and visualize. Can handle both numerical and categorical data. Captures non-linear relationships well.	Prone to overfitting, especially with deep trees. Sensitive to small changes in data, which can lead to different tree structures.
Support Vector Machine (SVM)	supervised learning model that finds the hyperplane that best separates classes in a high-dimensional space.	Effective in high-dimensional spaces. Works well with a clear margin of separation. Robust against overfitting in high dimensions.	Less interpretable than simpler models. Computationally intensive, especially with large datasets. Performance can be poor with overlapping classes.
Naïve Bayes (NB)	probabilistic classifier based on Bayes’ theorem, assuming independence between predictors	Simple and efficient, especially with large datasets. Works well with high-dimensional data. Performs well even with small amounts of data.	Assumes independence among features, which is often not the case in real-world data. Can be less accurate when this assumption is violated.
K-Nearest Neighbors (KNN)	non-parametric method that classifies instances based on the majority class of their k-nearest neighbors in the feature space	Simple and intuitive. No training phase; makes predictions directly from the dataset. Naturally handles multi-class problems.	Computationally expensive at prediction time, especially with large datasets. Sensitive to irrelevant features and the choice of k. Struggles with imbalanced datasets.
XGBoost (Extreme Gradient Boosting)	ensemble learning technique based on decision trees, designed for speed and performance.	High performance and accuracy. Handles missing values well and can capture complex patterns. Regularization helps prevent overfitting.	More complex and less interpretable than single models. Requires careful tuning of hyperparameters for optimal performance.
Artificial Neural Networks (ANN)	computational models inspired by the human brain, consisting of interconnected nodes (neurons) organized in layers.	Highly flexible and capable of modeling complex relationships. Can learn from large amounts of data and improve performance with more data. Effective for tasks involving unstructured data (e.g., images).	Requires substantial computational resources and time for training. Can be seen as a “black box,” making interpretation difficult. Prone to overfitting without proper regularization techniques.

Regarding the model sensitivity (recall), the NB and SVM models showed the lowest FN rate (highest sensitivity). It is important to consider trade-offs Between Accuracy and Sensitivity in interpreting the results of different classifiers. While overall accuracy is important, it can be misleading if the dataset is imbalanced (e.g., many infants do not require treatment). In such cases, a model with high accuracy might still fail to identify a significant number of at-risk infants. The choice of model should align with clinical priorities. If the goal is to minimize missed diagnoses (false negatives), then selecting a model with high sensitivity—even if it sacrifices some overall accuracy—makes sense. In medical settings, sensitivity (or recall) is critical because it measures a model’s ability to correctly identify patients who need treatment. A high sensitivity reduces the risk of missing cases that require intervention, which is especially important in high-stakes scenarios like neonatal care. So, from a clinical point of view, it may be more appropriate to choose a model with the lowest FN rate than a model with the highest accuracy. In other words, a model with high false positives does not pose a risk to patients. Although we emphasize the importance of sensitivity in predicting treatment-needed ROP, we recognize that the current integration of clinical insights into the model development process could be improved.

Furthermore, AI models must augment clinical judgment by providing actionable insights that clinicians can use in decision-making. In the context of ROP, a model with high sensitivity can serve as a supportive tool, prompting further investigation or intervention in borderline cases where clinical signs are ambiguous. We envision future iterations of the model being optimized with clinical input, not only focusing on sensitivity and specificity but also incorporating real-time feedback from clinicians to improve practical applicability. This will ensure that the models serve as reliable aids in clinical workflows, helping practitioners make more informed, data-driven decisions.

### Limitations and future scope

This study had some limitations. A major limitation of our study is that the data were obtained from a single center, which inherently limits the generalizability of our findings to other populations and clinical settings. Neonatal care practices, including oxygen therapy protocols, may vary across institutions and geographical regions, potentially affecting the model’s performance when applied elsewhere. Additionally, key features such as detailed information on oxygen therapy duration and intensity, maternal health factors, and other perinatal influences were not included in the dataset. These variables are known to have a significant impact on the risk of developing treatment-needed retinopathy of prematurity (ROP) and could improve the predictive power of the model. The absence of external validation across multiple centers further limits the reliability of our results. To ensure that the model is robust and generalizable, future research should incorporate multicenter datasets and include a broader range of clinical features. While AI can enhance decision-making processes by providing real-time risk assessments, these tools must be used to augment—not replace—clinical judgment. Clinicians must remain involved in interpreting model outputs and making final treatment decisions based on a holistic understanding of each patient’s unique circumstances.

Future research should focus on external validation using multicenter datasets and include additional clinical features that may impact the development of treatment-needed ROP. Moreover, exploring advanced machine learning techniques, such as ensemble models or deep learning, could improve sensitivity and overall model performance, enhancing clinical decision support in neonatal care.

## Conclusion

In our study, we developed and evaluated several supervised ML classifiers to predict treatment-needed ROP based on demographic and clinical data. While AI can enhance decision-making processes by providing real-time risk assessments, these tools must be used to augment—not replace—clinical judgment. Clinicians must remain involved in interpreting model outputs and making final treatment decisions based on a holistic understanding of each patient’s unique circumstances.

## Data Availability

The data supporting this study’s findings are available from the corresponding author upon reasonable request.

## Code availability

All codes are available at https://github.com/MehrdadMotamed-lab/ROP-prediction-models/blob/main/ROP_ADASYN.ipynb.

## Abbreviations

ROP: Retinopathy of prematurity
AUC: Area under the curve
LR: Logistic regression
DT: Decision tree
SVM: Support vector machine
NB: Naïve Bayes
KNN: K-nearest neighbors
ANN: Artificial neural networks
RF: Random forest
